# Anaerobic lignocellulolytic microbial consortium derived from termite gut: enrichment, lignocellulose degradation and community dynamics

**DOI:** 10.1186/s13068-018-1282-x

**Published:** 2018-10-17

**Authors:** Adèle Lazuka, Lucas Auer, Michael O’Donohue, Guillermina Hernandez-Raquet

**Affiliations:** Laboratoire d’Ingénierie des Systèmes Biologiques et des Procédés - LISBP, UMR5504, UMR792, CNRS, INRA, INSA, Université de Toulouse, 135 Avenue de Rangueil, 31077 Toulouse Cedex 04, France

**Keywords:** Lignocellulose, Anaerobic microbial consortium, Termite gut microbiome, Carboxylates, Xylanase, Cellulase

## Abstract

**Background:**

Lignocellulose is the most abundant renewable carbon resource that can be used for biofuels and commodity chemicals production. The ability of complex microbial communities present in natural environments that are specialized in biomass deconstruction can be exploited to develop lignocellulose bioconversion processes. Termites are among the most abundant insects on earth and play an important role in lignocellulose decomposition. Although their digestive microbiome is recognized as a potential reservoir of microorganisms producing lignocellulolytic enzymes, the potential to enrich and maintain the lignocellulolytic activity of microbial consortia derived from termite gut useful for lignocellulose biorefinery has not been assessed. Here, we assessed the possibility of enriching a microbial consortium from termite gut and maintaining its lignocellulose degradation ability in controlled anaerobic bioreactors.

**Results:**

We enriched a termite gut-derived consortium able to transform lignocellulose into carboxylates under anaerobic conditions. To assess the impact of substrate natural microbiome on the enrichment and the maintenance of termite gut microbiome, the enrichment process was performed using both sterilized and non-sterilized straw. The enrichment process was carried out in bioreactors operating under industrially relevant aseptic conditions. Two termite gut-derived microbial consortia were obtained from *Nasutitermes ephratae* by sequential batch culture on raw wheat straw as the sole carbon source. Analysis of substrate loss, carboxylate production and microbial diversity showed that regardless of the substrate sterility, the diversity of communities selected by the enrichment process strongly changed compared to that observed in the termite gut. Nevertheless, the community obtained on sterile straw displayed higher lignocellulose degradation capacity; it showed a high xylanase activity and an initial preference for hemicellulose.

**Conclusions:**

This study demonstrates that it is possible to enrich and maintain a microbial consortium derived from termite gut microbiome in controlled anaerobic bioreactors, producing useful carboxylates from raw biomass. Our results suggest that the microbial community is shaped both by the substrate and the conditions that prevail during enrichment. However, when aseptic conditions are applied, it is also affected by the biotic pressure exerted by microorganisms naturally present in the substrate and in the surrounding environment. Besides the efficient lignocellulolytic consortium enriched in this study, our results revealed high levels of xylanase activity that can now be further explored for enzyme identification and overexpression for biorefinery purposes.

**Electronic supplementary material:**

The online version of this article (10.1186/s13068-018-1282-x) contains supplementary material, which is available to authorized users.

## Background

Lignocellulose (LC) is the major component of the plant cell walls and constitutes the most abundant biomass on Earth. As such, LC is a renewable carbon resource that can be used to produce energy and commodity chemicals. A survey of natural ecosystems in which lignocellulose decomposition occurs reveals that the digestive systems of herbivores are particularly efficient [1, 2]. To digest biomass, herbivores benefit from symbiotic relationships with microbial communities that have been selected by millennial evolutionary processes [2]. Among the herbivores, termites are of particular interest since their digestive systems can be likened to highly efficient lignocellulolytic microscale bioreactors [1, 2].

Termites are among the most abundant insects on Earth [3]. They play an important role in the decomposition of plant material and global carbon recycling. Over the last ~ 150 million years, termites have developed various symbiotic strategies to digest lignocellulosic material, including highly lignified hardwood [4]. All termite species have in common that their guts contain microbial symbionts that contribute to nitrogen regulation and deliver fermentative products from biomass, such as acetate, propionate and other volatile fatty acids (VFA or carboxylates), as carbon and energy sources for their termite host [1, 2]. In return, the microbiome benefits from a stable environment and supply of nutrients.

In higher termites, such as the wood-feeding subfamily Nasutermitinae, the digestive tract can be described as a “dual cellulose digestion system”, consisting of three major compartments—the foregut, midgut and hindgut [5]. The foregut and midgut can be seen as small enzymatic bioreactors, where highly alkaline conditions and host-endogenous cellulases partially hydrolyze cellulose [6]. In contrast, the hindgut is more voluminous and usually displays a neutral or slightly acidic pH (pH 6–7). In the hindgut, hemicellulose and residual cellulose are metabolized by a symbiotic microbiome [2]. Indeed, recent investigations on *Nasutitermes* spp. showed that up to 50% of total cellulase activity in the hindgut was produced by the gut microbiome [7]. It is within the hindgut paunch that lignocellulolytic bacteria degrade plant fiber, deploying a rich diversity of carbohydrate-active enzymes to do so [8, 9]. For these reasons, the study of termite gut microbiomes is of considerable interest, in particular for biorefinery applications. Selecting microbial consortia from these environments, which naturally produce VFA as end products instead of converting them into methane, represents an opportunity to improve the carboxylate platform.

To reveal the biomass degradation potential of termite gut microbiomes and to identify the microbial species and enzymes involved in such processes, previous studies have applied culture-dependent approaches, enabling the isolation of cellulolytic and hemicellulolytic bacteria belonging to different taxa including *Bacteroides* and *Enterobacteriaceae* [10–12]. More recently, culture-independent “omics” methodologies have provided new insight into the composition of termite microbiomes and revealed a vast diversity of carbohydrate-active enzymes [2, 8, 9, 13]. The diversity of termite gut microbiome seems to vary in function of the diet and phylogeny of termites, but a dominance of phyla Spirochaetes, Bacteroidetes, Fibrobacteres, Firmicutes and the candidate phylum Termite Group 3 (TG3) has been noted [2]. Using a metagenomics approach, Warnecke et al. [8] revealed that the hindgut paunch of *Nasutitermes* species was mainly constituted by phylotypes related to the genus *Treponema* (Spirochaetes phylum) and the phylum Fibrobacteres; these phylotypes were identified as implicated on lignocellulose degradation as they code for diverse endoglucanases as well as for domains related to the catalytic site of glycoside hydrolases. Firmicutes also participate in lignocellulose transformation as they code numerous glycoside hydrolases belonging to the family 11 (GH11) [9].

In the biorefinery context, there is strong interest in the ability of microbial consortia to transform raw lignocellulosic biomass into valuable products such as methane, hydrogen or volatile fatty acids [14, 15]. To this end, efficient microbial consortia displaying target bioconversion features have been obtained by sequential enrichment culture [16, 17] and different inoculates, including microorganisms from soils [18–21], compost [16, 22–26] and cow rumen [27, 28]. However, despite the renowned potency of termite digestive systems, the use of these to artificially generate lignocellulolytic consortia has so far attracted little attention.

The aim of this study was to assess the *Nasutitermes ephratae* gut microbiome as a source of inoculum for an enrichment strategy on wheat straw to produce VFA. Previously, we have established that the microbiome from this species can be used to efficiently degrade wheat straw in bioreactors [29]. Therefore, in this study we set out to enrich and stabilize this wheat straw-degrading ability using a sequencing batch reactor (SBR) approach, operating under strict anaerobic conditions. To assess the impact of the endogenous microflora of raw substrate on the selection and maintenance of a termite-derived consortium, the enrichment processes were performed using both sterile (SS) and non-sterile (NSS) wheat straw as sole carbon sources. To simulate conditions that could be applied at industrial scale, bioreactors were operated under aseptic conditions. The microbial diversity during the enrichment process using both SS and NSS was characterized by 16S rRNA gene sequencing.

## Results

### Impact of substrate sterilization on enrichment of microbial consortia

**Fig. 1:**
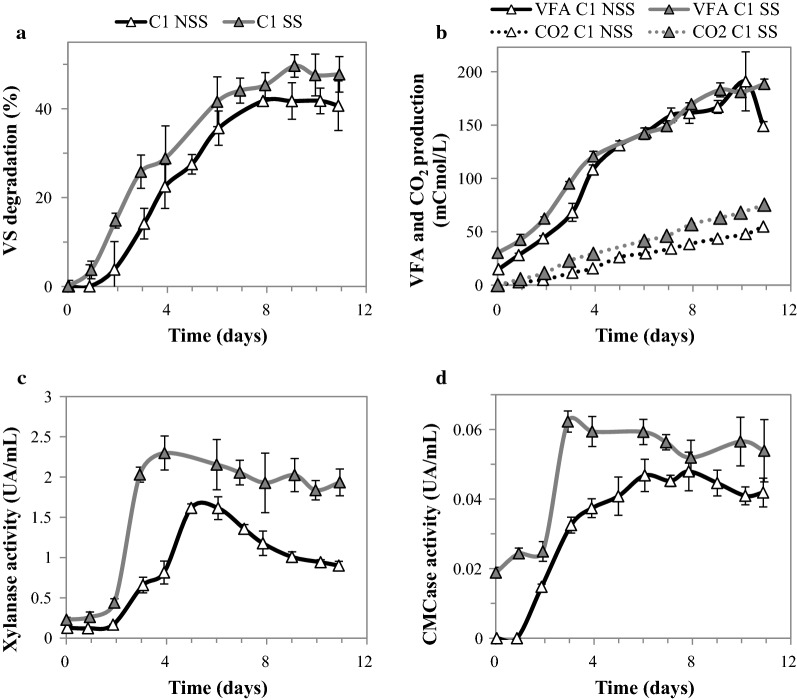
Characterization of the first cycle of enrichment (C1) of TM on SS and NSS. **a** VS degradation, **b** VFA production, **c** xylanase and **d** CMCase activities

Sequencing batch reactor enrichment has been previously used to successfully select more efficient lignocellulose-degrading consortia [17, 18, 28]. Therefore, to enrich the lignocellulolytic function of the *N. ephratae*-derived inoculum (hereafter called termite microbiome or TM), it was cultured in SBR mode using both milled sterile (SS) and non-sterile (NSS) raw wheat straw as sole carbon source. To simulate conditions that can be found at industrial scale, all enrichment experiments were performed in aseptic bioreactors. As the initial TM was produced on sterile substrate in two independent bioreactors (TMa and TMb), the first cycle of enrichment (C1) in both substrates was compared to assess the impact of the eventual differences in TM community composition, the endogenous wheat straw microbiome and the aseptic bioreactor conditions on biomass degradation (Fig. 1).

From an early phase of incubation, a different behavior of TM in each substrate (sterilized or not) was quite patent, because after only 3 days of incubation significantly higher levels of substrate degradation and VFA production were observed when using SS (C1 SS, 25.8 ± 3.7% VS and 95.2 ± 1.9 mCmol VFA L^−1^) compared to experiments with NSS (C1 NSS, 14.1 ± 3.4% VS and 68.2 ± 8.5 mCmol VFA L^−1^) (Fig. 1a, b). Likewise, at the end of incubation, the overall wheat straw degradation (VS) was higher in the case of bioreactors containing SS compared to those containing NSS. However, at the end-point VFA production was similar for both substrates (Fig. 1a, b). Taking into account that TOC measurements confirmed the absence of metabolites other than VFA in the fermentation broth, the difference in conversion yield (i.e., wheat straw to carboxylates) can be correlated with the significantly higher CO_2_ production observed in reactors containing SS compared to those containing NSS (Fig. 1b).

The effect of substrate sterility on key lignocellulolytic enzymes was assessed measuring xylanase and CMCase activities (Fig. 1c, d). In the reactor containing SS, a higher xylanase activity was measured, with maximum activity (2.3 UA mL^−1^) being observed at day 4. In the case of NSS, maximum xylanase activity was reached after day 5 and was 30% lower than that observed on SS. Thereafter, xylanase activity decreased in all the bioreactors, but a sharper decline was observed in those containing NSS compared to SS. Similarly, the maximum CMCase activity was higher and occurred earlier when the microbial consortium was grown on SS compared to NSS. However, unlike the xylanase activity, once maximal CMCase activity was achieved it remained quite stable over time, irrespective of the substrate used.

During the first cycle of TM enrichment on wheat straw, lignocellulose degradation was on average 40.7 ± 4.0% and 47.4 ± 5.0% of VS when growing on NSS and SS, respectively. The production of VFA followed a similar tendency, with similar production (190 mCmol VFA L^−1^) in all cultures. Since these results are representative of the initial LC degradation potential of TM, it was expected that they would constitute a basis for further functional enhancement through SBR enrichment.

**Fig. 2:**
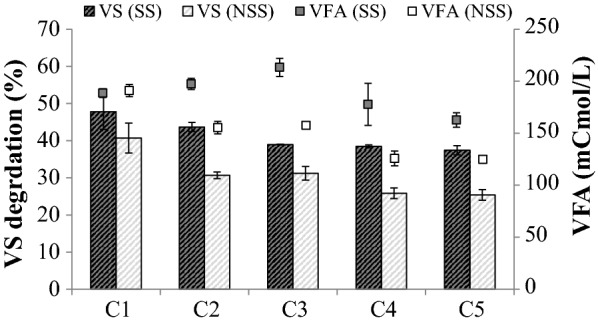
Effect of substrate sterility on wheat straw degradation (VS, bars) and carboxylate production (VFA, squares) during the enrichment process of termite-derived microbiome. SS and NSS indicate sterile and non-sterile substrate, respectively

In the case of the cultures growing on NSS, subsequent enrichment cycles revealed that lignocellulolytic capability declined, dropping to 30.7 ± 0.9% of VS at the end of the second cycle, while VFA production decreased by 20% (Fig. 2, C2). At the end of the SBR enrichment process, cultures growing on NSS displayed a potential for VS degradation and VFA production that were approximately 30% lower than those observed after the first cycle. In the case of cultures growing on SS, the decline in VS degradation and VFA production was much less pronounced (Fig. 2). After five enrichment cycles on SS, VS degradation had stabilized at 37.4 ± 1.2% VS and VFA production at around 167 ± 1.08 mCmol-VFA L^−1^. Therefore, although enrichment on SS did not reinforce the initial lignocellulolytic activity, it stabilized and was maintained at a high level (80% and 88% of the initial VS degradation and VFA production, respectively). The stabilized consortium produced using SS was thereafter referred to as TWS.

Bacterial diversity was studied by 16S rRNA gene sequencing in both sterilized and non-sterilized straw reactors. Simpson and Shannon indexes (Table 1) were very close between replicates, and showed a strong decrease at Cycle 1 for both sterilized and non-sterilized substrates. Thereafter, the diversity index stabilized at slightly higher values. There were no significant differences between the diversity index estimated for enrichments performed with sterilized or non-sterilized straw.

**Table 1:** Diversity index at each cycle of enrichment with sterile or non-sterile straw

Substrate	Cycle	Diversity index		
		Richness	Shannon	Simpson
SS	Gut	680	4.8	45
	TMa	125	2.8	8.5
	C1	118	1.1	2.0
	C2	119 ± 8	2 ± 0.03	4 ± 0.1
	C3	132 ± 5	2 ± 0.1	5 ± 0.03
	C4	109 ± 11	2 ± 0.1	5 ± 0.2
	C5	121 ± 6	1.9 ± 0.1	4 ± 0.3
NSS	Gut	587	4.8	44
	TMb	116	2.6	8.5
	C1	164	1.6	2.0
	C2	123 ± 1	1.9 ± 0.1	3 ± 0.1
	C3	122 ± 1	2 ± 0.07	4 ± 0.2
	C4	119 ± 7	2 ± 0.07	4 ± 0.4
	C5	127 ± 10	1.9 ± 0.03	4 ± 0.3

**Fig. 3:**
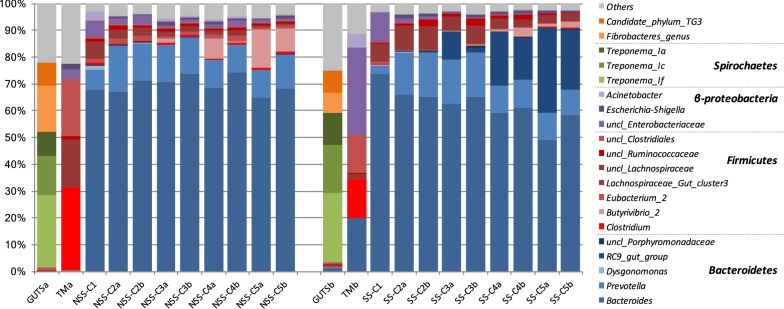
Diversity evolution during five cycles of enrichment starting with *N. ephratae* microbiome (TM) with non-sterile (left) and sterile straw (right). Cycles from one to five were performed in duplicate bioreactors (a and b) under aseptic conditions. The diversity of the initial termite gut is also shown (GUTS)

The diversity profiles showed that the initial termite gut inocula, dominated by *Spirochaetes* and *Fibrobacteres*, shifted after the first culture on wheat straw (TM), being largely dominated by *Firmicutes* (Fig. 3). Weighted UniFrac distance of OTUs (Additional file 1) showed that while the initial communities were close (C1 NSS or SS), they diverged throughout the enrichment process. Particularly, the communities developed on SS at the latest cycles of enrichment (C4 and C5) were clearly distinct to those observed on NSS, being more distant to the C1 community. Analyzing in more detail the community composition, from Cycle 1, the abundance of members related to *Bacteroides* and *Prevotella* genus (Bacteroidetes phyla) strongly increased at the expense of Firmicutes, with both SS and NSS wheat straw. Subsequent enrichment cycles presented a similar, stable diversity profile, with small variations of Bacteroidetes and Firmicutes content. During the enrichment processes, it is remarkable that termite-derived communities were significantly distant from the initial termite gut inocula. Nevertheless, in the final enriched communities (Cycle 3 to Cycle 5), some genera were specific to a given type of straw. *Butyrivibrio* reached 10% when non-sterile straw was used, whereas its abundance was much lower in sterilized straw bioreactors. Inversely, unclassified *Lachnospiraceae* and *Rikenellaceae RC9* genera (RC9 gut group) were more abundant when SS was used.

### Characterization of wheat straw termite-enriched microbiome TWS

**Fig. 4:**
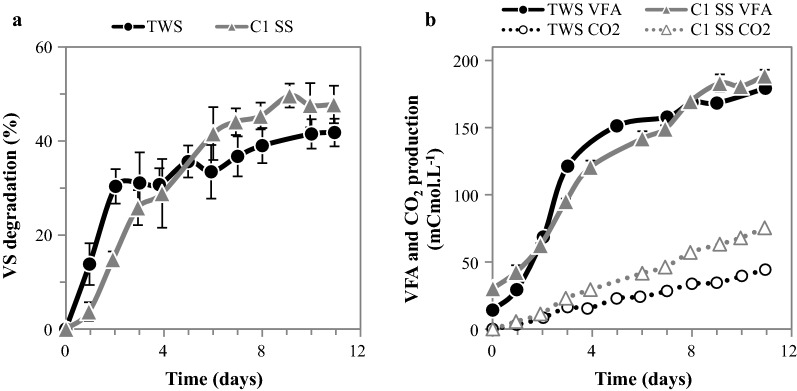
Comparing lignocellulose degradation and metabolites production by the TWS consortium and the first cycle of enrichment in sterile substrate (C1 SS). **a** VS degradation and **b** VFA and CO_2_ production

**Fig. 5:**
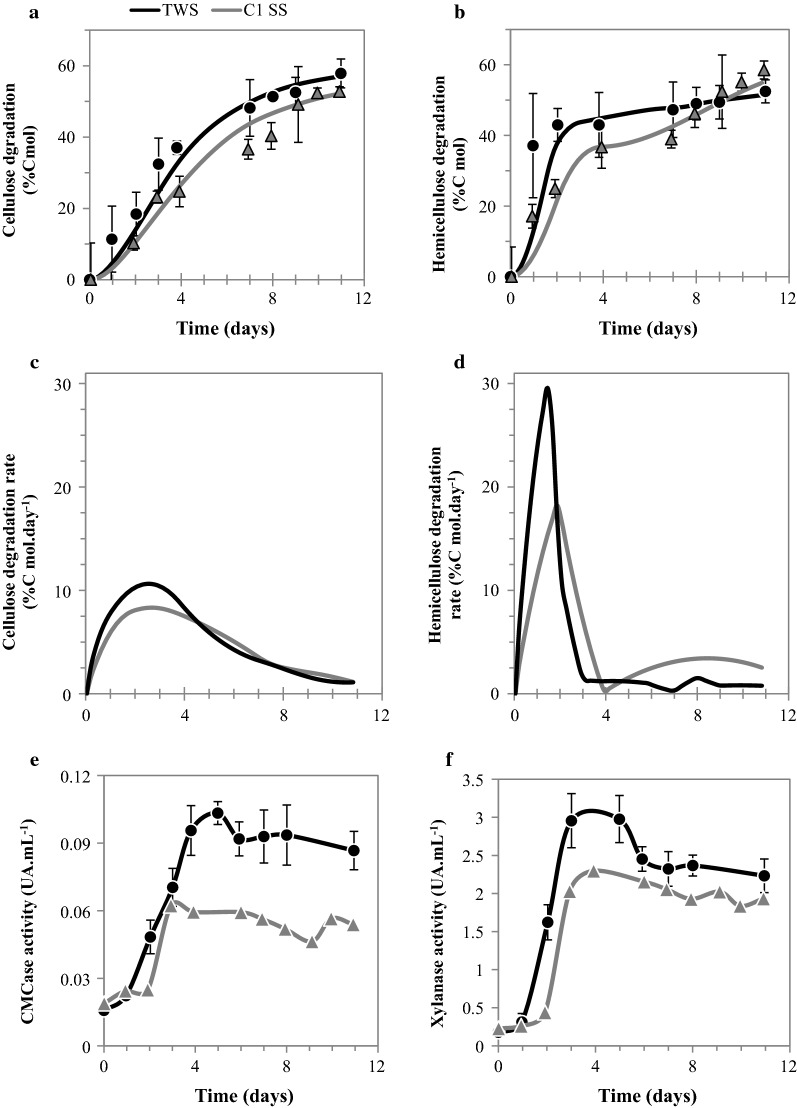
Comparison of the activity of the TWS consortium and the first cycle of enrichment in sterile substrate (C1 SS) on cellulose and hemicellulose fractions of wheat straw. **a** Cellulose and **b** hemicellulose degradation; **c** cellulose and **d** hemicellulose degradation rate, and **e** CMCase and **f** xylanase activities

The kinetic behavior of TWS enriched on sterile straw was investigated, monitoring substrate degradation, carboxylate production, and major enzyme activities. To assess the benefit of the enrichment process, TWS data were compared to those obtained during the first enrichment cycle on SS (C1 SS; Fig. 4). The stabilized community TWS achieved on average 41.8 ± 2.9% VS degradation and a production of 179.3 ± 3.1 mCmol VFA L^−1^ after 11 days incubation, representing about 87–95% of the respective values observed in C1 SS (Fig. 4a, b). The main differences between C1 SS and TWS were observed during the early phase of incubation, with TWS displaying higher lignocellulose degradation and VFA production rates compared to C1 SS. Indeed, at day 2, substrate degradation of TWS was twice that measured in C1 SS (30.4 and 14.8% VS degradation, respectively). Similarly, TWS displayed a higher VFA production during the first days of incubation compared to C1 SS (about 158.0 vs 120.6 mCmol VFA L^−1^ on day 4, respectively). TWS also displayed a greater ability to remove hemicellulose compared to C1 SS (Fig. 5b), with the maximal degradation rate being observed in day 1 (29.6% Cmol L^−1^ day^−1^), whereas C1 SS displayed a lower value (17.0% Cmol L^−1^ day^−1^) that was reached on day 2 (Fig. 5d). Overall, the hemicellulose degradation efficiency of TWS was enhanced by the enrichment process.

Regarding cellulose degradation, although C1 SS and TWS displayed similar global cellulose degradation profiles (Fig. 5a), examination of the early phase (day 3) revealed that TWS attained 20% higher cellulose degradation rate compared to C1 SS (Fig. 5c). Similarly, on day 2, cellulose degradation by TWS was 80% higher than that achieved by C1 SS (Fig. 5a). Overall, it is possible to conclude that TWS preferentially degraded hemicellulose, although both cellulose and hemicellulose degradation were enhanced and occurred sooner than in the first enrichment cycle (C1 SS). Likewise, the data indicate that hemicellulose hydrolysis sharply declined after day 4, whereas cellulose degradation declined progressively over a longer period of time up to day 10 (Fig. 5a–d). This observation is even more pronounced for TWS community compared to C1 SS.

Regarding the dynamics of enzyme activities, both CMCase (endoglucanase activity) and xylanase activities (Fig. 5e, f) displayed profiles composed of three distinct phases. After a short lag phase, both activities sharply increased over an initial period, reaching a maximum, and then decreased slowly. The maximal values achieved for CMCase and xylanase activities were 60% and 22%, respectively, and were higher for TWS. It is noteworthy that while cellulose degradation and CMCase activity reached maximum values at the same time in the case of both C1 SS and TWS, the higher CMCase activity observed in TWS did not correlate with higher cellulose degradation. This is probably related to the availability and recalcitrance of the residual cellulose. In contrast, the hemicellulose degradation rates observed in reactors C1 SS and TWS correlated well with increased xylanase activity. Moreover, in TWS a higher xylanase activity corresponded to a higher hemicellulose degradation rate. This is apparently the main benefit of enrichment of the TWS consortium.

**Fig. 6:**
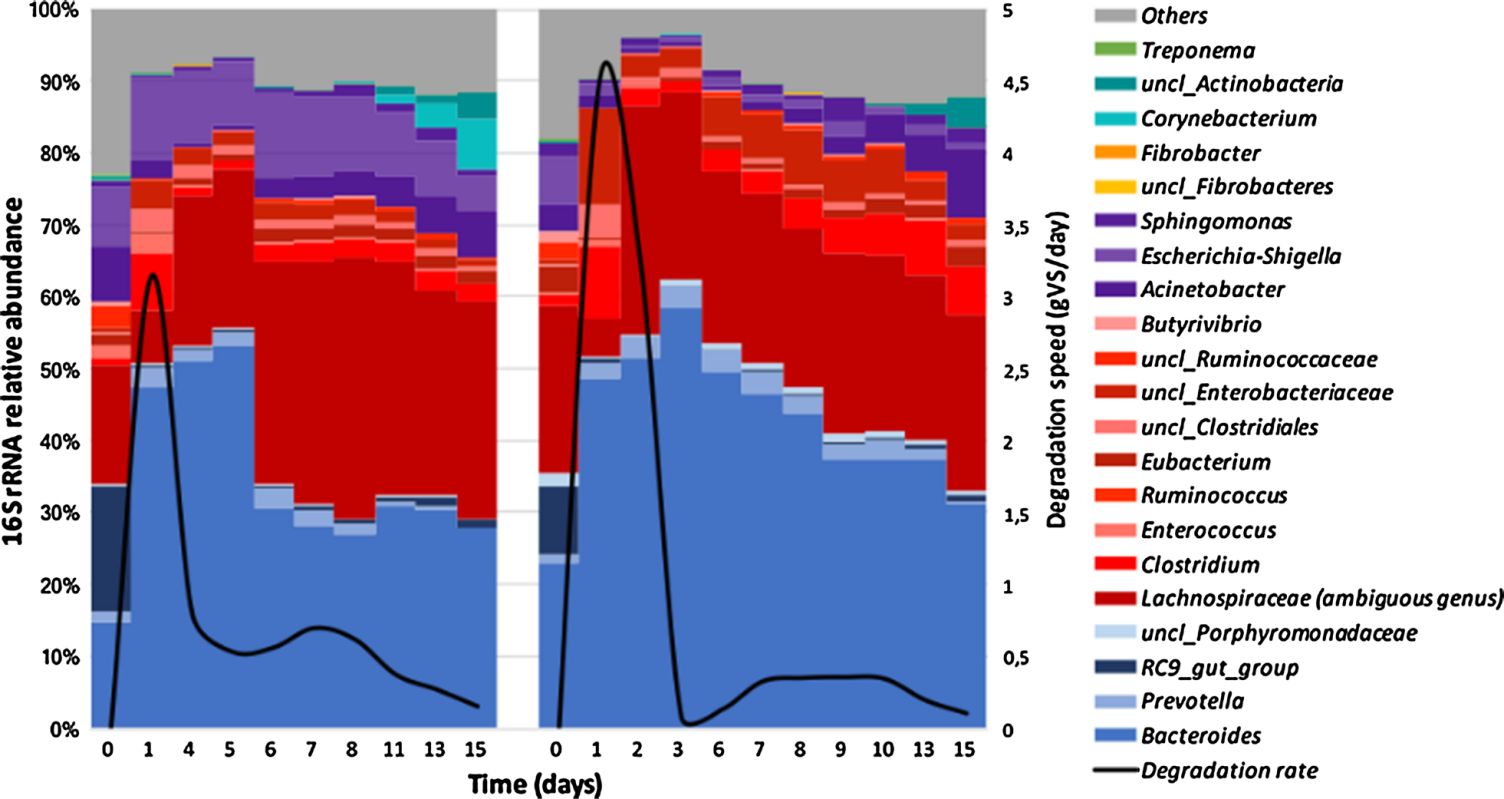
Diversity profiles of 16S rRNA throughout lignocellulose degradation by TWS in the two replicate bioreactors (bioreactor 1—left; bioreactor 2—right). Numbers in X-axis correspond to days. Colors correspond to bacterial phyla *Bacteroidetes* (blue), *Firmicutes* (red), *Proteobacteria* (purple), *Fibrobacteres* (orange), *Actinobacteria* (cyan) and *Spirochaetes* (green). Degradation rate is indicated by the black curves

To correlate the lignocellulose degradation ability of TWS to its diversity, we studied diversity changes throughout a lignocellulose degradation cycle (Fig. 6). Monitoring the active TWS community by sequencing 16S rRNA transcripts revealed that *Bacteroides* became highly prevalent over time and that progression of this genus correlated well with lignocellulose degradation. Indeed, at the highest lignocellulose degradation rate, *Bacteroides* and *Lachnospiraceae* accounted for 70–85% of the total 16S rRNA content. Thereafter, the abundance of *Lachnospiraceae* stabilized, whereas *Bacteroides* decreased, being replaced by Proteobacteria. Sparse PLS-discriminant analysis confirmed that *Bacteroides graminisolvens* formed the OTU that most highly correlated with lignocellulose degradation (Additional file 2), followed by a *Prevotella* OTU. Other OTUs belonging to *Eubacterium* and *Acinetobacter* genus were associated with the plateau phase of degradation. ANOVA analysis of the remaining OTUs did not reveal significant differences in their abundance between the peak and plateau phases. The major differences being explained by *Bacteroides*, *Acinetobacter*, *Eubacterium* and *Prevotella* related OTUs.

## Discussion

### Impact of substrate sterilization on microbial consortia enrichment

In the SBR enrichment process, bacterial selection is based on microbial growth capacity, selecting microorganisms on their ability to grow faster under defined conditions. In the present case, the key determinants were the ability to grow on wheat straw as the sole carbon source and outcompete natural microflora present on wheat straw, or in the surrounding environment (i.e., in the case of bioreactors carried out under aseptic conditions). Irrespective of substrate sterility, we observed that the initial microbial diversity present in the termite gut was radically altered during the enrichment process on wheat straw, implying that the substrate and the aseptic, anaerobic conditions of the bioreactor constituted the major determinant of the final outcome. Indeed, the communities developed during the C1 cycle on both NSS and SS, displayed very similar composition with a high abundance of Bacteroidetes (mainly *Bacteroides* and *Prevotella* members). In all bioreactors, this phylum replaced the Firmicutes members that were present in the original TM inocula. In this respect it is noteworthy that, although culturing was performed under strictly sterile condition, the community composition of TM was already different from that of the actual termite gut, which is mainly constituted of Spirochaetes and Fibrobacteres. Further enrichment cycles under aseptic conditions resulted in stronger diversity changes that were accompanied by a decrease in lignocellulose degradation capacity and VFA production, particularly when NSS was used as substrate. Presumably, these changes can be attributed to faster growth of microorganisms that possess the greatest adaptability to the prevailing conditions, meaning that Spirochaetes and Fibrobacteres present in the termite gut and Firmicutes members present in TM were not among these. Significantly, the Bacteroidetes and Firmicutes that became abundant during the enrichment process only represented minor components of the original termite gut microflora. The appearance of these was probably promoted by their ability to consume the available nutrients. In this respect, it is noteworthy that the genera *Bacteroides* and *Prevotella,* enriched during the cultivation process on wheat straw, are known to be lignocellulose degraders that possess high growth rates and thus possess significant advantages over the slower growing microorganisms in the unstabilized community [30, 31]. It is also plausible that microorganisms present in the environment or the endogenous microflora of wheat straw (in the case of NSS) exerted strong pressure on the original termite gut microbiome, preventing its full establishment in the bioreactors. The progressive decline in the aptitude of the consortium grown on NSS to degrade lignocellulose seems to be the result of changes in microbial diversity. This was previously suggested by Reddy et al. [26] and Lazuka et al. [28], who reported strong changes in microbial diversity during the enrichment process. This in turn would explain the decline of xylanase production, which obviously impacts lignocellulose degradation. In the case of NSS, the wheat straw endogenous bacteria probably competed with the lignocellulolytic microorganisms present in the original inoculum, resulting in the faster decline of lignocellulose degradation capability in the subsequent enrichment cycles compared to SS. Several studies have shown a strong “substrate effect” on community composition [32, 33], which drives community selection. Therefore, it is unsurprising that the enrichment process on wheat straw generated similarities (at species/OTU level) between the communities developed on NSS and SS bioreactors. Nevertheless, diversity analysis also revealed differences between the NSS- and SS-related communities, which can be correlated with dissimilar lignocellulose-degrading capacities. Indeed, although high abundances of *Bacteroides* and *Prevotella* were observed in both substrates throughout the enrichment processes, the abundance of *Butyrivibrio* genus was higher in non-sterile straw, while unclassified *Lachnospiraceae* and *Rikenellaceae* RC9 were more abundant in the enrichment with sterile straw. These two families were dominated by unique OTUs (i.e., OTU_3_ and OTU_2_). In the case of OTU_3_, it presents 100% identity to a novel Clostridia isolated from a biogas reactor and described as a cellulose degrader, which has temporarily been classified as *Clostridium* (GenBank: LN868251.1; unpublished). Numerous species of *Clostridium* have previously been reported as displaying cellulase and hemicellulose activities [25, 34, 35]. Similarly, OTU_2_ presents 100% identity with an uncultured fiber-associated rumen bacteria [36]. Rikenellaceae is a not well-described family with only two genera identified as displaying a tolerance to growth under acidic conditions and presenting an anaerobic metabolism. Therefore, these albeit sparse data correlate with the higher lignocellulose degradation levels observed in SS compared to NSS bioreactors.

It is noteworthy that a previous study focused on a cellulose-degrading community (H-C) derived from soil, Feng et al. [18] found that substrate sterilization had little impact on the outcome of the enrichment process. Using autoclaved and non-autoclaved corn stover (milled 40 mesh = 375 µm size), the authors reported degradation levels of 52.8% and 50.8%, respectively. However, unlike in the present study, it is noteworthy that before testing the consortium on non-sterile corn stover, Feng et al. [18] performed enrichment exclusively on a sterile substrate (autoclaved filter paper). This implies that the enriched consortium was already stabilized before it was confronted with a non-sterile substrate. These observations have important implications for enriching lignocellulolytic communities from particular environments such as termite gut: to enrich such communities successfully, they should be protected as much as possible from other microorganisms such as those naturally present in wheat straw or in the surrounding environment.

### Characterization of TWS enriched in sterilized wheat straw

The diversity of the most efficient termite-derived consortium TWS, obtained after five cycles of enrichment on sterilized wheat straw, was rather low, with mainly Bacteroidetes members belonging to four main genera, representing up to 95% of the community composition. Such strong selection could be explained by the loss of the major part of the initial inoculum, unable to grow fast enough in the prevailing fermentation conditions. Indeed, bioreactor conditions are far from the conditions prevailing in termite gut where the host consumes free sugars and VFA [2]. Here, while the bioreactor’s pH was rather similar to that found in *Nasutitermes* gut [37], bioreactors are perfectly mixed systems which differ from the conditions prevailing in termite gut; other factors such as unsatisfied nutrient or O_2_ requirements, altered host relationship or inter-taxa dependency might also explain the differences observed with respect to the initial inoculum. Termites are known to be very efficient lignocellulose degraders, displaying between 74 and 99% cellulose and 65 and 87% hemicellulose degradation rates [2]. Such degradation levels are high compared to those observed in TWS enrichment which displayed 57.8% cellulose and 52.4% hemicellulose degradation levels. The difference observed between the diversity of termite gut inoculum [29], the shift of diversity during the enrichment process and the final TWS diversity might explain the lower lignocellulose degradation rates finally observed in TWS. It also suggests that, by modifying the culture conditions in bioreactors, it could be possible to increase the lignocellulose degradation performances to attain maximal termite gut capacities. Nevertheless, LC degradation and VFA production capabilities of the enriched consortium TWS were higher or comparable to those previously reported using artificial ecosystems. For example, using a rumen fluid inoculum, Chang et al. [27] observed 11% degradation of raw napier grass (initial concentration 1.5% w/v). Another study that employed a soil inoculum growing in aerobic conditions reported degradation levels of 47.7, 43.8 and 43.3% when growing on 1% (w/v) switchgrass, corn stover and wheat straw, respectively [21]. However, this study did not report VFA production levels. Similarly, using a soil-enriched consortium growing at 40 °C under static conditions (i.e., not strict anaerobic), Feng et al. [18] reported 51% degradation of a fine (40 mesh) corn stover powder (1% w/v initial concentration). In this case, substrate degradation was accompanied by a very low VFA production, probably due to the non-strict anaerobic conditions applied. Using a compost-derived microbial consortium (XDC-2), Hui et al. [38] measured 39.6%, 25.2% and 17.6% degradation of 1% (w/v) rice straw, wheat straw and corn stover, respectively. Finally, in a recent study Sheng et al. [39] reported the enrichment of a microbial consortium derived from *Holotrichia parallela* (dark black chafer) that rapidly and efficiently (83% degradation over a 3-day period, 1% w/v initial concentration) degraded NaOH-pretreated rice straw. However, metabolite production was not monitored. In summary, it is quite difficult to compare results from different studies, because substrates, substrate pretreatment and culture conditions are generally different. Moreover, in previous studies the aim was often the production of enzymes rather than metabolites, such as carboxylates. Nevertheless, considering the initial substrate concentration reported in these studies, the best performance was reported by Sheng et al. (8.3 g L^−1^ rice straw degradation in 3 days) using a alkali-pretreated rice straw [39]. In the same period, TWS degraded 6.2 g L^−1^ of un-pretreated wheat straw. We can conclude that the lignocellulolytic capability of TWS is comparatively high.

In this study, substrate sterilization has been identified as a positive factor for lignocellulose degradation. However, it is important to note that autoclaving might have modified the substrate, increasing its degradability [40]. Nevertheless, considering the relative lability of the different components, the dry conditions applied and the low temperature employed for substrate sterilization (121 °C) were relatively mild and would not have engendered significant structural or chemical modifications [41, 42]. For example, Lawther et al. [43] showed that steam treatment at 120 °C during 15–300 min did not have a significant impact on the chemical composition of wheat straw. Similarly, Garrote et al. [41] demonstrated that temperatures above 200 °C were needed to degrade cellulose by steam treatment. In the present study, as autoclaving was carried out under dry conditions, it could be expected that the eventual impact of autoclaving, mainly driven by water ionization [41], would be very weak. Therefore, it appears reasonable to mostly attribute increased hemicellulose degradation to the reinforcement of this function during the enrichment process, which favored xylanase activity in TWS. Similar results have been reported by Guo et al. [22], who observed that specific hemicellulose degradation was correlated with increased xylanase activity (maximum 8.454 U mL^−1^) with an enriched soil consortium (XDC-2). XDC-2 removed 77.1% of rice straw hemicellulose after 3 days, but the cellulose fraction was poorly degraded (11.2%, progressing to 36.7% removal after 12 days), with a constant low level of CMCase activity (0.5 UA mL^−1^). Compared to XDC-2, the degree of hemicellulose degradation by TWS was significantly lower (52.4% vs 77.1%, respectively), but TWS also degraded cellulose (57.9%), which is the main polysaccharide in the biomass. Interestingly, this function of TWS was the result of a CMCase activity that was about one-fifth of the maximum activity measured in the XDC-2 experiment. The XDC-2 consortium has also been tested on raw wheat straw [38]. In this case, a poor degradation level was achieved (25.2%), which compares unfavorably with the TWS consortium. From an industrial standpoint, TWS produces enzymes active on both cellulose and hemicellulose fractions of wheat straw and TWS operates in anaerobic conditions, thus obviating the need for aeration.

In a previous study, we have reported the enrichment of an anaerobic rumen-derived microbial consortium, RWS, which displayed good lignocellulose degradation capability and VFA production on raw wheat straw [28, 44]. When RWS was grown on NaOH-pretreated wheat straw, high xylanase (1.2 UA mL^−1^) and CMCase (0.05 UA mL^−1^) activities were measured. Therefore, it is noteworthy that TWS achieved higher or comparable levels of enzyme activity (2.9 UA mL^−1^ for xylanase and 0.05 UA mL^−1^ for CMCase) when acting on raw wheat straw. Consistently, TWS displayed a higher hemicellulose degradation rate (29.6% per day) than RWS (14.6% per day). These results strongly suggest that TWS is a better purveyor of xylanase and, to a lesser extent, CMC-degrading enzymes compared to RWS.

Compared to other enriched lignocellulolytic communities [21, 23, 26–28], the rather simple community profile of TWS allows the establishment of putative correlations between degradation parameters and specific OTUs. Considering the 16S rRNA transcript levels as an indicator of bacterial activity, two OTUs, OTU_1_ (*Bacteroides*) and OTU_3_ (*Lachnospiraceae*, potentially *Clostridium*), accounting for up to 80% of the 16S rRNA relative abundance in the system, appeared extremely active. The temporal dynamics of OTU_1_ were well correlated with maximum lignocellulose degradation and xylanase activity (until a plateau was reached). Therefore, OTU_1_ appears to be responsible for the strong hemicellulose degradation observed at the initial phase of incubation. In contrast, OTU_3_ did not display any correlation with wheat straw degradation, but it correlated well with CMCase activity, suggesting its implication in cellulose degradation. Although the enzyme activity measurements performed reflect the overall enzymatic potential of the system, they do not enable the identification of the individual enzymes involved, nor do they provide the means to relate them to specific microbial taxa. Nevertheless, given that Firmicutes are known to produce a larger repertoire of carbohydrate-active enzymes (CAZymes) than Bacteroidetes [45], it is plausible that there is interplay between *Bacteroides* and *Lachnospiraceae*, which were constantly present throughout wheat straw degradation. However, further meta-transcriptomic or meta-proteomic studies would be required to validate this.

In conclusion, although it was not feasible to enrich the original diversity of *N. ephratae* gut microbiome in anaerobic bioreactors, we successfully enriched and stabilized a lignocellulolytic community TWS using sterile wheat straw in aseptic conditions. This microbial consortium efficiently degraded 42% of raw lignocellulose within 12 days and presented an initial preference for hemicellulose. Wheat straw was mainly converted into VFA. During enrichment, xylanase and CMCase activities increased possibly thanks to the enrichment of OTUs belonging to *Bacteroides* and *Lachnospiraceae*, respectively. Overall, TWS is a potentially interesting reservoir for lignocellulolytic enzymes and a prototype for further research to explore its enzymatic system and to optimize a process for carboxylate production from biomass.

## Methods

### Lignocellulosic substrate and inoculum

20 kg of wheat straw (winter wheat variety Koreli) was harvested and milled to 2 mm as described by Lazuka et al. [28]. One kilogram of this raw substrate, designated non-sterile straw (NSS), was autoclaved under dry conditions (120 °C, 20 min, 1.1 bar), yielding sterile straw (SS).

The initial termite gut inoculum (500 dissected guts) from *N. ephratae* was provided by IRD (Institute for Research and Development, Bondy, France). This was used to inoculate an anaerobic bioreactor containing autoclaved wheat straw (120 °C, 15 min, 1.1 bar) as sole carbon source, as described by Auer et al. [29]. At the end of the incubation period (20 days), the resulting culture was snap frozen in liquid nitrogen and stored at − 80 °C. This inoculum is hereafter designated as termite-derived microbiome (TM).

### Wheat straw anaerobic bioreactors

Microbial cultures were carried out in anaerobic bioreactors (2 L BIOSTAT^®^ A+, Sartorius, Germany) using mineral medium (MM) and wheat straw (NSS or SS) as the sole carbon source (20 g L^−1^) according to the conditions detailed by Lazuka et al. [28]. MM contained per liter of distilled water: KH_2_PO_4_, 0.45 g, K_2_HPO_4_, 0.45 g; NH_4_Cl, 0.4 g; NaCl, 0.9 g; MgCl_2_·6H_2_O, 0.15 g; CaCl_2_·2H_2_O, 0.09 g. MM was supplemented with 250 μL of V7 vitamin solution [46] and 1 mL trace elements solution, containing per liter of distilled water: H_3_BO_3_, 300 mg; FeSO_4_·7H_2_O, 1.1 g; CoCl_2_·6H_2_O, 190 mg; MnCl_2_·4H_2_O, 50 mg; ZnCl_2_, 42 mg; NiCl_2_·6H_2_O, 24 mg; NaMoO_4_·2H_2_O, 18 mg; CuCl_2_·2H_2_O, 2 mg; sterilized by filtration (0.2 µm). The reactors were flushed with nitrogen after inoculation; the absence of dissolved oxygen was monitored with a polarographic dissolved oxygen probe (AppliSens). Bioreactors were operated at constant temperature (35 °C) and pH (6.15), the latter being controlled by the addition of an alkali solution (1 M NaOH).

To progressively enrich LC-degrading microbial consortia, two parallel batch bioreactors were inoculated (10% v/v) with TM (thawed overnight at 4 °C) and fed with SS or NSS as the sole carbon source. These two reactors, representing the first enrichment cycle (SS C1 and NSS C1), were operated until VFA production was stable. Subsequent enrichment cycles were inoculated (10% v/v) with a fraction from the previous cycle and were incubated over 11-day periods using SS or NSS as the carbon source. After the first enrichment cycle, duplicate experiments (two SS C2 reactors and two NSS C2, respectively) using either substrate were initiated by the inoculation (10% v/v) of two parallel bioreactors with the culture obtained from the relevant C1 cycle. After five enrichment cycles, VFA production was stabilized. At this stage, samples (200 mL) of termite-enriched consortia were snap frozen under liquid nitrogen and stored at − 80 °C. To simulate conditions that can be found at industrial scale, enrichment experiments were carried under aseptic conditions; before use, bioreactors were cleaned by immersion in a concentrated bleach solution.

To characterize the dynamics of LC degradation by the highest performing microbial consortium (i.e., the one obtained through enrichment on sterile straw), samples were thawed overnight at 4 °C and used as inocula for further experiments. To further characterize LC degradation by this consortium, bioreactors were operated as biological duplicates for 11 days using operating conditions identical to those described above. To assess the impact of enrichment on LC degradation, the kinetics of the culture were compared to those of the initial culture (i.e., SS C1).

### Chemical analyses

Total solids (TS) were determined by centrifuging (7197×*g*, 10 min) a sample (10 mL) removed from bioreactors. The solid pellet was rinsed twice with distilled water and dried at 105 °C for 24 h. The mineral fraction (MF) was determined by mineralization of the samples at 500 °C for 2 h, and volatile solids (VS) were estimated by subtracting TS from MF. VS degradation was expressed as weight/weight (w/w) percentages.

Wheat straw composition was determined using the sulfuric acid hydrolysis method described by de Souza et al. [47] and modified by Lazuka et al. [28]. Monomeric sugar concentration was determined using the HPLC analytical protocol described by Monlau et al. [48] and an Ultimate 3000 Dionex separation system equipped with a BioRad Aminex HPX 87H affinity column and a refractive index detector (Thermo Scientific).

Volatile fatty acids production was determined by gas chromatography (GC), using a Varian 3900 chromatograph as described by Cavaillé et al. [49]. The total organic carbon (TOC) content of the liquid fraction was measured using a TOC analyzer (TOC-V_CSN_, Shimadzu). Gas composition was analyzed using a chromatograph HP 5890 equipped with a conductivity detector and a HAYSEP D column.

All of the macro-kinetic parameters were expressed as average values obtained in duplicate biological reactors, except for the first enrichment cycle where average values were obtained from technical duplicate samples.

### Enzymatic activities

For enzyme activity measurements, samples (5 mL) were withdrawn at regular intervals from the bioreactors and supernatants and solid pellets were obtained after centrifugation as described by Lazuka et al. [28]. Supernatants were correlated with extracellular enzyme activity, whereas that measured after suspension and sonication of solid pellets was correlated with total cell-bound enzymes. For each bioreactor and each sampling time, end-point enzymatic activities were measured in technical duplicates in both the extracellular and cell-bound fractions. Enzymatic activities were expressed as average values obtained on duplicate bioreactors.

Xylanase and endoglucanase (CMCase) activities were measured using 1% w/v xylan beechwood (Sigma) and 1% w/v carboxymethyl cellulose (CMC) (Sigma) in the conditions described by Lazuka et al. [28]. One unit of CMCase or xylanase activity (UA, unit of activity) was defined as the amount of enzyme that produces 1 µmol of reducing sugars per minute.

### Diversity analysis

Diversity was assessed at each final point during the enrichment processes and at each sampling point (approximatively daily) for the kinetic characterization of bioreactors. 1.5 mL samples were taken and immediately centrifuged (13,000×*g*, 5 min and 4 °C), the supernatant was removed and the pellet was frozen in liquid nitrogen. Samples were stored at − 80 °C until nucleic acid extraction. Total DNA and RNA were extracted using a PowerMicrobiome RNA Isolation kit (MoBio Laboratories Inc. Carlsbad) following the manufacturer’s instructions, but omitting the final DNAse steps. Cell lysis was carried out with a Fast Prep (MP Biomedicals) (2 × 30 s at 4 ms^−1^). Subsequently, DNA and RNA were separated and purified using an AllPrep DNA/RNA Mini Kit (Qiagen) according to the manufacturer’s instructions. DNA and RNA integrity and purity were checked by agarose gel (1%) electrophoresis. Concentrations were measured by NanoDrop 1000 spectrophotometer (Thermo Scientific), measuring absorbance at 260 and 280 nm. Residual DNA content in RNA samples was removed using 1 µg RNA and a DNAse (TURBO DNA-free™ Ambion, Life Technologies) according to the manufacturer’s instructions. RNA was then retro-transcripted into cDNA using M-MLV Reverse Transcriptase (Promega) and random hexamers (Roche) following the manufacturer’s instructions. Sequencing of the V3–V4 region of 16S rRNA gene was performed after PCR amplification using the bacterial primers 343F and 784R, modified to add sequencing adaptors during a second PCR (343F = 5′-CTT TCC CTA CAC GAC GCT CTT CCG ATC TAC GGR AGG CAG CAG-3′ and 784R = 5′-GGA GTT CAG ACG TGT GCT CTT CCG ATC TTA CCA GGG TAT CTA ATC CT-3′). The library was prepared as previously detailed [50] and loaded on a MiSeq Illumina cartridge, using reagent kit v3 (paired 300 bp reads). Sequencing was performed at the GenoToul Genomics and Transcriptomics facility (GeT, Auzeville, France) using a MiSeq^®^ Illumina^®^.

### Diversity data processing

Data were demultiplexed and pair-end reads were joined by the GeT platform, using Flash v1.2.6 [51], 110 bp minimum overlap and a 0.1 maximum mismatches ratio. Fastq files were transformed into a unique fasta file and treated with mothur v1.33.1 [52] following the SRF1 procedure described in Auer et al. [50]. A fusion of LTP SSU database, version 115 [53], and DictDB [54], a database dedicated to insect-associated bacteria, was used to improve taxonomic assignation of sequences from termite gut-derived communities. Phylogenetic trees were constructed using ClustalO [55] and raxmlHPC [56]. OTU tables, taxonomic files and phylogenetic trees were imported into Phyloseq package v1.14.0 [57] using R v3.2.3, following the manual import procedure. Shannon and Simpson indexes were evaluated using Phyloseq functions. Partial least square (PLS) analyses were performed using mixOmics v5.2.0 package [58]. ANOVA tests were performed using basic R functions anova() and lm().

Sequencing data are available at 10.15454/XTIHB5 (not yet publicly accessible, for now see https://data.inra.fr/privateurl.xhtml?token=03130738-7eec-4758-965e-ed7780af53f7).

## Additional files

**Additional file 1.** Clustered tree and PCoA plot based on weighted Unifrac distance.

**Additional file 2.** Sparse PLS-DA applied to degradation peak and degradation plateau points.

## References

[1] Godon JJ, Arcemisbéhère L, Escudié R, Harmand J, Miambi E, Steyer JP (2013). Overview of the oldest existing set of substrate-optimized anaerobic processes: digestive tracts. Bioenergy Res.

[2] Brune A (2014). Symbiotic digestion of lignocellulose in termite guts. Nat Rev Microbiol.

[3] Kambhampati S, Eggleton P, Abe T, Bignell DA, Higashi M (2000). Taxonomy and phylogenetics of isoptera. Termites: evolution, sociality, symbioses and ecology.

[4] Konig H, Li L, Frohlich J (2013). The cellulolytic system of the termite gut. Appl Microbiol Biotechnol.

[5] Nakashima K, Watanabe H, Saitoh H, Tokuda G, Azuma JI (2002). Dual cellulose-digesting system of the wood-feeding termite, *Coptotermes formosanus* Shiraki. Insect Biochem Mol Biol.

[6] Tokuda G, Watanabe H, Hojo M, Fujita A, Makiya H, Miyagi M, Arakawa G, Arioka M (2012). Cellulolytic environment in the midgut of the wood-feeding higher termite *Nasutitermes takasagoensis*. J Insect Physiol.

[7] Tokuda G, Watanabe H, Lo N (2007). Does correlation of cellulase gene expression and cellulolytic activity in the gut of termite suggest synergistic collaboration of cellulases?. Gene.

[8] Warnecke F, Luginbuhl P, Ivanova N, Ghassemian M, Richardson TH, Stege JT, Cayouette M, McHardy AC, Djordjevic G, Aboushadi N, Sorek R, Tringe SG, Podar M, Martin HG, Kunin V, Dalevi D, Madejska J, Kirton E, Platt D, Szeto E, Salamov A, Barry K, Mikhailova N, Kyrpides NC, Matson EG, Ottesen EA, Zhang X, Hernandez M, Murillo C, Acosta LG, Rigoutsos I, Tamayo G, Green BD, Chang C, Rubin EM, Mathur EJ, Robertson DE, Hugenholtz P, Leadbetter JR (2007). Metagenomic and functional analysis of hindgut microbiota of a wood-feeding higher termite. Nature.

[9] He S, Ivanova N, Kirton E, Allgaier M, Bergin C, Scheffrahn RH, Kyrpides NC, Warnecke F, Tringe SG, Hugenholtz P (2013). Comparative metagenomic and metatranscriptomic analysis of hindgut paunch microbiota in wood- and dung-feeding higher termites. PLoS ONE.

[10] Schultz JE, Breznak JA (1978). Heterotrophic bacteria present in hindguts of wood-eating termites [*Reticulitermes flavipes* (Kollar)]. Appl Environ Microbiol.

[11] Dheeran P, Nandhagopal N, Kumar S, Jaiswal YK, Adhikari DK (2012). A novel thermostable xylanase of *Paenibacillus macerans* IIPSP3 isolated from the termite gut. J Ind Microbiol Biotechnol.

[12] Azizi-Shotorkhoft A, Mohammadabadi T, Motamedi H, Chaji M, Fazaeli H (2016). Isolation and identification of termite gut symbiotic bacteria with lignocellulose-degrading potential, and their effects on the nutritive value for ruminants of some by-products. Anim Feed Sci Technol.

[13] Franco Cairo JP, Leonardo FC, Alvarez TM, Ribeiro DA, Buchli F, Costa-Leonardo AM, Carazzolle MF, Costa FF, Paes Leme AF, Pereira GA, Squina FM (2011). Functional characterization and target discovery of glycoside hydrolases from the digestome of the lower termite *Coptotermes gestroi*. Biotechnol Biofuels.

[14] Kleerebezem R, van Loosdrecht MC (2007). Mixed culture biotechnology for bioenergy production. Curr Opin Biotechnol.

[15] Agler MT, Wrenn BA, Zinder SH, Angenent LT (2011). Waste to bioproduct conversion with undefined mixed cultures: the carboxylate platform. Trends Biotechnol.

[16] Haruta S, Cui Z, Huang Z, Li M, Ishii M, Igarashi Y (2002). Construction of a stable microbial community with high cellulose-degradation ability. Appl Microbiol Biotechnol.

[17] Cheng YF, Edwards JE, Allison GG, Zhu WY, Theodorou MK (2009). Diversity and activity of enriched ruminal cultures of anaerobic fungi and methanogens grown together on lignocellulose in consecutive batch culture. Bioresour Technol.

[18] Feng Y, Yu Y, Wang X, Qu Y, Li D, He W, Kim BH (2011). Degradation of raw corn stover powder (RCSP) by an enriched microbial consortium and its community structure. Bioresour Technol.

[19] Zhou Y, Pope PB, Li S, Wen B, Tan F, Cheng S, Chen J, Yang J, Liu F, Lei X, Su Q, Zhou C, Zhao J, Dong X, Jin T, Zhou X, Yang S, Zhang G, Yang H, Wang J, Yang R, Eijsink VG, Wang J (2014). Omics-based interpretation of synergism in a soil-derived cellulose-degrading microbial community. Sci Rep.

[20] Jimenez DJ, Dini-Andreote F, van Elsas JD (2014). Metataxonomic profiling and prediction of functional behaviour of wheat straw degrading microbial consortia. Biotechnol Biofuels.

[21] de Lima Brossi MJ, Jimenez DJ, Cortes-Tolalpa L, van Elsas JD (2016). Soil-derived microbial consortia enriched with different plant biomass reveal distinct players acting in lignocellulose degradation. Microb Ecol.

[22] Guo P, Zhu W, Wang H, Lu Y, Wang X, Zheng D, Cui Z (2010). Functional characteristics and diversity of a novel lignocelluloses degrading composite microbial system with high xylanase activity. J Microbiol Biotechnol.

[23] Wongwilaiwalin S, Rattanachomsri U, Laothanachareon T, Eurwilaichitr L, Igarashi Y, Champreda V (2010). Analysis of a thermophilic lignocellulose degrading microbial consortium and multi-species lignocellulolytic enzyme system. Enzyme Microb Technol.

[24] Lin C-W, Wu C-H, Tran D-T, Shih M-C, Li W-H, Wu C-F (2011). Mixed culture fermentation from lignocellulosic materials using thermophilic lignocellulose-degrading anaerobes. Process Biochem.

[25] Wang W, Yan L, Cui Z, Gao Y, Wang Y, Jing R (2011). Characterization of a microbial consortium capable of degrading lignocellulose. Bioresour Technol.

[26] Reddy AP, Allgaier M, Singer SW, Hazen TC, Simmons BA, Hugenholtz P, VanderGheynst JS (2011). Bioenergy feedstock-specific enrichment of microbial populations during high-solids thermophilic deconstruction. Biotechnol Bioeng.

[27] Chang J-J, Lin J-J, Ho C-Y, Chin W-C, Huang C-C (2010). Establishment of rumen-mimic bacterial consortia: a functional union for bio-hydrogen production from cellulosic bioresource. Int J Hydrogen Energy.

[28] Lazuka A, Auer L, Bozonnet S, Morgavi DP, O’Donohue M, Hernandez-Raquet G (2015). Efficient anaerobic transformation of raw wheat straw by a robust cow rumen-derived microbial consortium. Bioresour Technol.

[29] Auer L, Lazuka A, Sillam-Dusses D, Miambi E, O’Donohue M, Hernandez-Raquet G (2017). Uncovering the potential of termite gut microbiome for lignocellulose bioconversion in anaerobic batch bioreactors. Front Microbiol.

[30] Nishiyama T, Ueki A, Kaku N, Watanabe K, Ueki K (2009). *Bacteroides graminisolvens* sp. nov., a xylanolytic anaerobe isolated from a methanogenic reactor treating cattle waste. Int J Syst Evol Microbiol.

[31] Stevenson DM, Weimer PJ (2007). Dominance of Prevotella and low abundance of classical ruminal bacterial species in the bovine rumen revealed by relative quantification real-time PCR. Appl Microbiol Biotechnol.

[32] Eichorst SA, Joshua C, Sathitsuksanoh N, Singh S, Simmons BA, Singer SW (2014). Substrate-specific development of thermophilic bacterial consortia by using chemically pretreated switchgrass. Appl Environ Microbiol.

[33] Simmons CW, Reddy AP, Simmons BA, Singer SW, VanderGheynst JS (2014). Effect of inoculum source on the enrichment of microbial communities on two lignocellulosic bioenergy crops under thermophilic and high-solids conditions. J Appl Microbiol.

[34] Thomas L, Joseph A, Gottumukkala LD (2014). Xylanase and cellulase systems of *Clostridium* sp.: an insight on molecular approaches for strain improvement. Bioresour Technol.

[35] Tracy BP, Jones SW, Fast AG, Indurthi DC, Papoutsakis ET (2012). Clostridia: the importance of their exceptional substrate and metabolite diversity for biofuel and biorefinery applications. Curr Opin Biotechnol.

[36] Zened A, Combes S, Cauquil L, Mariette J, Klopp C, Bouchez O, Troegeler-Meynadier A, Enjalbert F (2013). Microbial ecology of the rumen evaluated by 454 GS FLX pyrosequencing is affected by starch and oil supplementation of diets. FEMS Microbiol Ecol.

[37] Kohler T, Dietrich C, Scheffrahn RH, Brune A (2012). High-resolution analysis of gut environment and bacterial microbiota reveals functional compartmentation of the gut in wood-feeding higher termites (*Nasutitermes* spp.). Appl Environ Microbiol.

[38] Hui W, Jiajia L, Yucai L, Peng G, Xiaofen W, Kazuhiro M, Zongjun C (2013). Bioconversion of un-pretreated lignocellulosic materials by a microbial consortium XDC-2. Bioresour Technol.

[39] Sheng P, Huang J, Zhang Z, Wang D, Tian X, Ding J (2016). Construction and characterization of a cellulolytic consortium enriched from the hindgut of *Holotrichia parallela* larvae. Int J Mol Sci.

[40] Fengel D, Wegener G (1984). Wood—chemistry, ultrastructure, reactions. J Polym Sci Lett Ed.

[41] Garrote G, Domínguez H, Parajó JC (1999). Hydrothermal processing of lignocellulosic materials. Holz als Roh- und Wer.

[42] Hendriks AT, Zeeman G (2009). Pretreatments to enhance the digestibility of lignocellulosic biomass. Bioresour Technol.

[43] Lawther JM, Sun R, Banks WB (1996). Effect of steam treatment on the chemical composition of wheat straw. Holzforschung.

[44] Lazuka A, Roland C, Barakat A, Guillon F, O’Donohue M, Hernandez-Raquet G (2017). Ecofriendly lignocellulose pretreatment to enhance the carboxylate production of a rumen-derived microbial consortium. Bioresour Technol.

[45] El Kaoutari A, Armougom F, Gordon JI, Raoult D, Henrissat B (2013). The abundance and variety of carbohydrate-active enzymes in the human gut microbiota. Nat Rev Microbiol.

[46] Pfennig N, Trüper HG, Balows A, Trüper HG, Dworkin M, Harder W, Schleifer KH (1992). The family Chromatiaceae. The prokaryotes.

[47] de Souza AC, Rietkerk T, Selin CG, Lankhorst PP (2013). A robust and universal NMR method for the compositional analysis of polysaccharides. Carbohydr Polym.

[48] Monlau F, Barakat A, Steyer JP, Carrere H (2012). Comparison of seven types of thermo-chemical pretreatments on the structural features and anaerobic digestion of sunflower stalks. Bioresour Technol.

[49] Cavaille L, Grousseau E, Pocquet M, Lepeuple AS, Uribelarrea JL, Hernandez-Raquet G, Paul E (2013). Polyhydroxybutyrate production by direct use of waste activated sludge in phosphorus-limited fed-batch culture. Bioresour Technol.

[50] Auer L, Mariadassou M, O’Donohue M, Klopp C, Hernandez-Raquet G (2017). Analysis of large 16S rRNA Illumina data sets: impact of singleton read filtering on microbial community description. Mol Ecol Resour.

[51] Magoc T, Salzberg SL (2011). FLASH: fast length adjustment of short reads to improve genome assemblies. Bioinformatics.

[52] Schloss PD, Westcott SL, Ryabin T, Hall JR, Hartmann M, Hollister EB, Lesniewski RA, Oakley BB, Parks DH, Robinson CJ, Sahl JW, Stres B, Thallinger GG, Van Horn DJ, Weber CF (2009). Introducing mothur: open-source, platform-independent, community-supported software for describing and comparing microbial communities. Appl Environ Microbiol.

[53] Yarza P, Richter M, Peplies J, Euzeby J, Amann R, Schleifer KH, Ludwig W, Glockner FO, Rossello-Mora R (2008). The all-species living tree project: a 16S rRNA-based phylogenetic tree of all sequenced type strains. Syst Appl Microbiol.

[54] Mikaelyan A, Kohler T, Lampert N, Rohland J, Boga H, Meuser K, Brune A (2015). Classifying the bacterial gut microbiota of termites and cockroaches: a curated phylogenetic reference database (DictDb). Syst Appl Microbiol.

[55] Sievers F, Higgins DG (2014). Clustal Omega, accurate alignment of very large numbers of sequences. Methods Mol Biol.

[56] Stamatakis A (2006). RAxML-VI-HPC: maximum likelihood-based phylogenetic analyses with thousands of taxa and mixed models. Bioinformatics.

[57] McMurdie PJ, Holmes S (2013). phyloseq: an R package for reproducible interactive analysis and graphics of microbiome census data. PLoS ONE.

[58] Le Cao KA, Boitard S, Besse P (2011). Sparse PLS discriminant analysis: biologically relevant feature selection and graphical displays for multiclass problems. BMC Bioinform.

