# Supplementary material for: Anaerobic lignocellulolytic microbial consortium derived from termite gut: enrichment, lignocellulose degradation and community dynamics
Source: Biotechnol Biofuels. 2018 Oct 17;11:284. doi: 10.1186/s13068-018-1282-x (PMC6191919; doi:10.1186/s13068-018-1282-x)
Supplement: Supplementary file 1 — Additional file 1. Clustered tree and PCoA plot based on weighted Unifrac distance. [file 13068_2018_1282_MOESM1_ESM.docx]

Additional file 1

Clustered tree and PCoA plot based on weighted-Unifrac distance

Weighted-Unifrac distances were calculated using phyloseq R-package. Clustering was performed using hclust R function with ward.D2 parameter, and PCoA plot was performed using phyloseq ordinate and plot functions.
NSS and SS communities appear to separate with cycles: C1 are well grouped, C2 and C3 begin to be distant and C4 and C5 are well separated. On the PCoA plot, NSS-C5a appear to be close to C1 points, but it is in fact due to its projection according to the 3^rd^ and 4^th^ axes. Its position in the clustered tree and the raw distances confirm that it is distant from SS-C1 and NSS-C1.


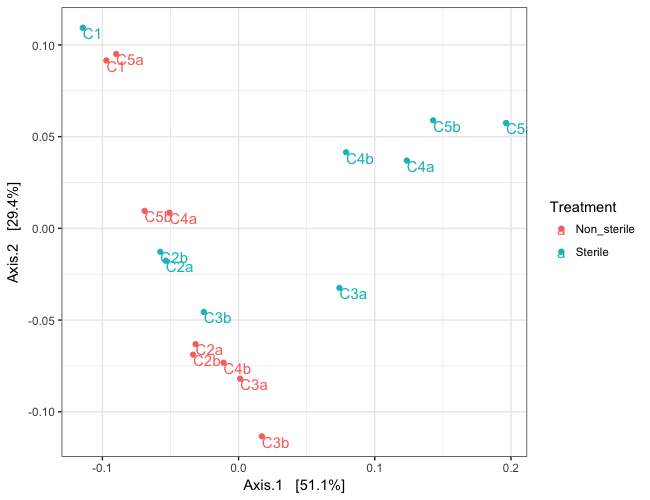

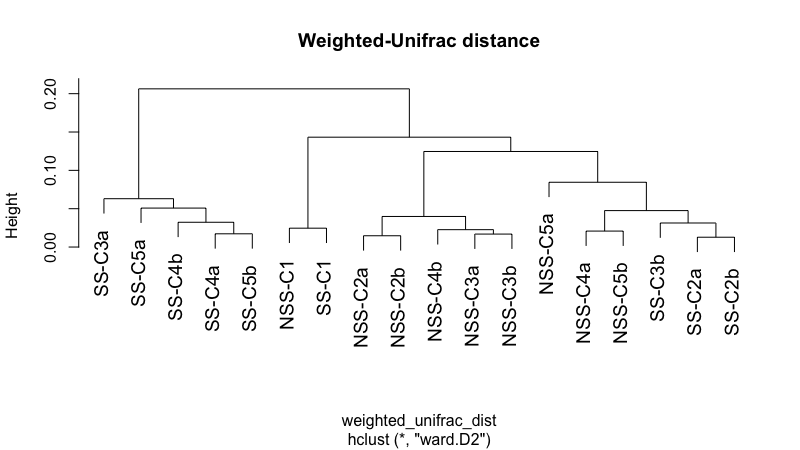


Weighted-Unifrac distances between samples
