# Supplementary material for: Anaerobic lignocellulolytic microbial consortium derived from termite gut: enrichment, lignocellulose degradation and community dynamics
Source: Biotechnol Biofuels. 2018 Oct 17;11:284. doi: 10.1186/s13068-018-1282-x (PMC6191919; doi:10.1186/s13068-018-1282-x)
Supplement: Supplementary file 2 — Additional file 2. Sparse PLS-DA applied to degradation peak and degradation plateau points. [file 13068_2018_1282_MOESM2_ESM.docx]

**Additional file 2**

Sparse PLS-DA applied to degradation peak and degradation plateau points.

On the left, sPLS-DA plot with colors representing replicates (blue for replicate 1 and orange replicate 2) and numbers corresponding to sample days. Days from 1 to 5 were attributed to Peak group while days from 8 to 13 were in Plateau group. On the right, correlation circle at the OTU level. Taxonomy of the selected OTUs and coordinates in Component 1 are detailed in the table below, with the corresponding ANOVA Pr-value based on OTU abundance in the two groups.

| **OTU** | **Component1 coordinates** | **ANOVA Pr-value** |
| --- | --- | --- |
| ***Eubacterium_2_Otu018*** | **0.4966** | **6.01e-05** |
| ***Acinetobacter_Otu005*** | **0.4823** | **1.43e-04** |
| ***Bacteroides_graminisolvens_Otu001*** | **-0.4032** | **2.48e-03** |
| *unclassified_Comamonadaceae_Otu039* | 0.2487 | 3.33e-02 |
| *Prevotella_Otu008* | -0.1637 | 7.83e-02 |
| *unclassified_Clostridiales_Otu015* | -0.1295 | 1.04e-01 |
| *unclassified_Lachnospiraceae_Otu003* | 0.0717 | 1.58e-01 |
| Others | 0.4974 | 5.70e-05 |

Relative abundances of main OTUs, based on rRNA sequencing data (next page).

Only peak and plateau points are shown. Only OTUs that displayed 2% abundance in at least one sampling point are shown, the others were gathered in “Others” category.

|  | C1a_T01 | C1a_T02 | C1a_T03 | C1a_T06 | C1a_T08 | C1b_T01 | C1b_T02 | C1b_T03 | C1b_T08 | C1b_T09 |
| --- | --- | --- | --- | --- | --- | --- | --- | --- | --- | --- |
| Bacteroidetes;Bacteroidia;Bacteroidales;Bacteroidaceae; Bacteroides;graminisolvens_Otu001 | 0.4142 | 0.4602 | 0.5123 | 0.2494 | 0.2878 | 0.4505 | 0.3452 | 0.4016 | 0.2995 | 0.3214 |
| Firmicutes;Clostridia;Clostridiales;Lachnospiraceae; unclassified;unclassified_Otu003 | 0.0734 | 0.2084 | 0.2181 | 0.3637 | 0.2871 | 0.055 | 0.3164 | 0.2577 | 0.2383 | 0.2268 |
| Bacteroidetes;Bacteroidia;Bacteroidales;Bacteroidaceae; Bacteroides;unclassified_Otu004 | 0.0485 | 0.0318 | 0.0123 | 0.0129 | 0.0131 | 0.0282 | 0.1633 | 0.1775 | 0.0658 | 0.0508 |
| Proteobacteria;Gammaproteobacteria;Enterobacteriales;Enterobacteriaceae; Escherichia-Shigella;unclassified_Otu009 | 0.1178 | 0.1054 | 0.0882 | 0.099 | 0.076 | 0.0144 | 0.0052 | 0.0055 | 0.0109 | 0.011 |
| Firmicutes;Clostridia;Clostridiales;Clostridiaceae; Clostridium;unclassified_Otu006 | 0.0752 | 0.0156 | 0.0145 | 0.0257 | 0.0277 | 0.101 | 0.0228 | 0.0134 | 0.0586 | 0.0776 |
| Proteobacteria;Gammaproteobacteria;Enterobacteriales;Enterobacteriaceae; unclassified;unclassified_Otu010 | 0.0394 | 0.0242 | 0.0182 | 0.0215 | 0.0141 | 0.1296 | 0.0288 | 0.0264 | 0.063 | 0.0298 |
| Proteobacteria;Gammaproteobacteria;Pseudomonadales;Moraxellaceae; Acinetobacter;unclassified_Otu005 | 0.0189 | 0.0046 | 0.006 | 0.0268 | 0.0482 | 0.0074 | 0.0044 | 0.0053 | 0.0291 | 0.0407 |
| Firmicutes;Clostridia;Clostridiales;unclassified; unclassified;unclassified_Otu015 | 0.0327 | 0.0126 | 0.0064 | 0.0074 | 0.0077 | 0.0498 | 0.014 | 0.0138 | 0.0073 | 0.0047 |
| Firmicutes;Clostridia;Clostridiales;Clostridiaceae; unclassified;unclassified_Otu030 | 0.0355 | 0.0053 | 0.0053 | 0.0094 | 0.0075 | 0.0438 | 0.0075 | 0.0026 | 0.0132 | 0.0109 |
| Bacteroidetes;Bacteroidia;Bacteroidales;Prevotellaceae; Prevotella;unclassified_Otu008 | 0.0226 | 0.0097 | 0.0108 | 0.0087 | 0.0006 | 0.0116 | 0.0245 | 0.0166 | 0.0129 | 0.0084 |
| Firmicutes;Clostridia;Clostridiales;Eubacteriaceae; Eubacterium_2;unclassified_Otu018 | 0.001 | 0.0116 | 0.0062 | 0.0215 | 0.0197 | 0.0009 | 0.0007 | 0.0064 | 0.02 | 0.016 |
| Proteobacteria;Alphaproteobacteria;Sphingomonadales;Sphingomonadaceae; Sphingomonas_3;unclassified_Otu026 | 0.0038 | 0.0058 | 0.0043 | 0.0228 | 0.0155 | 0.008 | 0.0121 | 0.0036 | 0.0019 | 0.0123 |
| Proteobacteria;Betaproteobacteria;Burkholderiales;Comamonadaceae; unclassified;unclassified_Otu039 | 0.0005 | 0.0041 | 0.0008 | 0.0014 | 0.0211 | 0.0015 | 0.0011 | 0.0006 | 0.0086 | 0.0152 |
| Firmicutes;Bacilli;Lactobacillales;unclassified; unclassified;unclassified_Otu007 | 0.0333 | 0.0015 | 0.0005 | 0.002 | 0.0016 | 0.0097 | 0.0023 | 0.0005 | 0.0015 | 0.0009 |
| Actinobacteria;Actinobacteria;Corynebacteriales;Corynebacteriaceae; Corynebacterium_8;unclassified_Otu019 | 0.0007 | 0.0008 | 0.0001 | 0.001 | 0.0359 | 0.0001 | 0 | 0.0002 | 0 | 0.0004 |
| Others | 0.0825 | 0.0984 | 0.096 | 0.1268 | 0.1364 | 0.0885 | 0.0517 | 0.0683 | 0.1694 | 0.1731 |
